# A case study on co-exposure to a mixture of organic solvents in a Tunisian adhesive-producing company

**DOI:** 10.1186/1745-6673-6-28

**Published:** 2011-11-14

**Authors:** Imed Gargouri, Moncef Khadhraoui, Catherine Nisse, Ariane Leroyer, Mohamed L Masmoudi, Paul Frimat, Daniel Marzin, Boubaker Elleuch, Denis Zmirou-Navier

**Affiliations:** 1 Water, Energy and Environment Laboratory, National school of Engineers, Sfax University, Sfax - Tunisia; 2 University laboratory of Occupational Medicine and Occupational Hazards, EA 2690 Poisons and occupational carcinogens and the environment. Medical School, Lille 2 University, Lille - France; 3 Department of Occupational Medicine and Professional Pathology, University Hospital/Medical School, Sfax University, Sfax - Tunisia; 4 Inserm U954 (National Institute for Health and Medical Research), Nancy - France; 5 School of Medicine, Nancy University, Vandoeuvre-lès-Nancy - France; 6 EHESP School of Public Health, Rennes - France

**Keywords:** Adhesive manufacturing, Organic solvents, Hexane, Bio-monitoring, Occupational exposure assessment

## Abstract

**Objectives:**

to assess environmental and biological monitoring of exposure to organic solvents in a glue-manufacturing company in Sfax, Tunisia.

**Methods:**

Exposure of volunteer workers, in the solvented glue-work-stations, in the control laboratory and in the storage rooms of the finished products, was assessed through indoor-air and urine measurements. Informed consent of the workers was obtained.

**Results and discussion:**

The exposure indexes were found with high values in the solvented workshop as well as in the control laboratory and were respectively, 8.40 and 3.12. These indexes were also correlated with hexane and toluene indoor air concentrations. As to urine, the obtained results for the 2,5-hexandione and hippuric acid, metabolites of hexane and toluene, respectively, were in accord with the indoor-air measurements, with an average of 0.46 mg/l and 1240 mg/g of creatinine.

**Conclusion:**

This study assessed for the first time biological exposure to organic solvents used in Tunisian adhesive industries. Although values are likely to underestimate true exposure levels, some figures exceed European and American occupational exposure guidelines.

## Introduction

Organic solvents are a group of mainly volatile compounds widely used to dissolve other substances in industrial processes among them adhesive manufacturing ones. Through their handling, these solvents can be released into the environment during production, storage, transportation and utilisation [1,2]. Being volatile, they can quickly evaporate and thus might be found with high concentrations in the air. They are consequently commonly inhaled in their volatilized form and absorbed via the respiratory tract [3-5]. They can also penetrate deeply though the skin in case of a direct contact.

Among the chemical risks listed in the Tunisian adhesive producing companies, organic solvents occupy by far the first place [4,6]. However in spite of the large quantity being used, there is little information on exposure and on the adverse health effects solvents may cause.

Sfax, the second largest town in Tunisia after the capital Tunis, is located further to the South. It is one of the industrial and agricultural pillars of the Tunisian economy. Among its industries, adhesive manufacturing companies are one of the main industrial bases in the region. Generally speaking, their products are used in various fields such as shoe making, a very prosperous activity in the area.

Prevention of occupational hazards, and more particularly of chemical risks, is usually based on regulatory procedures [2,7]. The Tunisian regulation on health in the workplace however does not require employers to carry out atmospheric exposure measurements nor to assess biological exposure indexes (BEI) [8-10]. This investigation was therefore conducted as a set up of occupational exposure study at an adhesive manufacturing company where the mainly used solvents are acetone, cyclohexane, n-hexane, methylethylketone, toluene and trichloroethylene. The investigation aimed to assess occupational exposure of the company volunteer's workers to a mixture of solvents in order to improve the plant working conditions.

## Material and methods

### Presentation of the company

The adhesive company was created in the 1960's. Currently, it consists of 5 work stations, 2 storage rooms for the raw materials and finished products, a control laboratory and an office. The total staffs are composed of 45 employees: 7 administrators, 2 engineers, 5 technicians, 29 workers and 2 drivers. The 5 production lines correspond to the workshops of: dissolved, natural, latex, powder and vinyl adhesives. The company is characterized by the stability of its staff and the absence of a significant modification in the manufacturing process and therefore of occupational tasks since 2000. We assume therefore that solvents exposure has been quite steady for a long time. The work schedule is 8 hours/day and 42 hours/week.

**Figure 1:**
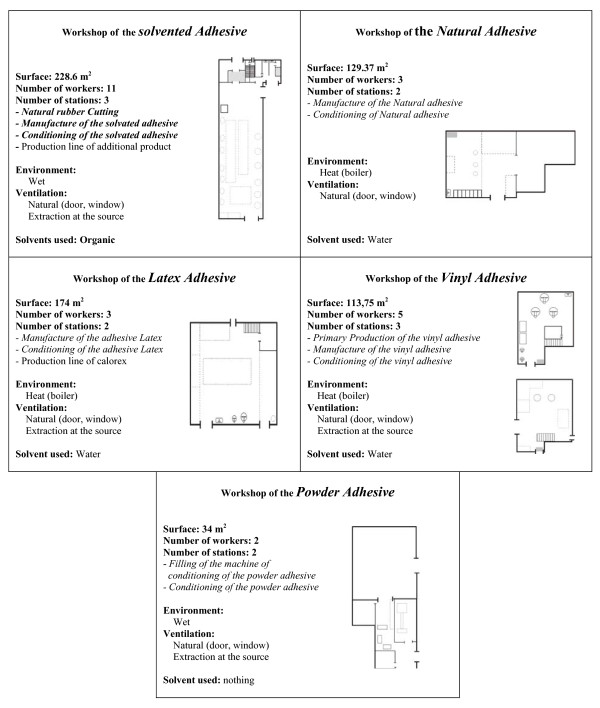
**Description of the various workshops**. Workshop of the solvented Adhesive: Rubber, Polychloroprene, Polyurethane and Produced additional.

Regarding the company production area, it is made of 5 workshops (Figure 1) according to the nature of the raw material of adhesives. Each workshop has its own characteristics; however in general, it is composed of 2 principal sections. In the first one, adhesives of different types are produced, with a maximum number of 3 operators. Conditioning takes place in the second section where the number of permanent operators varies from 2 to 7 according to the workshop and the quantity of the produced adhesive. It is worth noting that under some circumstances; in particular in case of very important orders in one of the workshops, some employees might move transitorily from one workshop to another. In these workshops, various chemicals are handled for adhesives manufacturing, among them organic solvents whose daily and annual quantities are listed in Table 1.

**Table 1:** Quantities of solvents used in the company and chemical identification

Solvents	Number		Quantities (m^3^) during	
	
	CAS n°	EINECS	Year (%)	Day of air sampling
Acetone*	67-64-1	200-662-2	102.5 (17.5)	0.5
Butyl acetate	123-86-4	204-658-1	2.5 (0.4)	-
Cyclohexane*	110-82-7	203-806-2	57.8 (9.9)	0.6
Ethyl acetate	141-78-6	205-500-4	33.5 (5.7)	-
n-hexane*	110-54-3	203-777-6	120.0 (20.5)	2.0
Methylethylcetone*	78-93-3	201-159-0	150.0 (25.6)	1.7
Perchloroethylene	127-18-4	204-825-9	2.0 (0.3)	-
Toluene*	108-88-3	203-625-9	116.0 (19.8)	1.5
Trichloroethylene	79-01-6	201-167-4	0.7 (0.1)	-

*: solvents handled during the of air sampling period

### Study population

Twenty five employees who were working in the manufacturing and conditioning lines, in the control laboratory and in the storage halls of the finished products (10 men, 15 women, median age 44.8 years [min = 25, max = 58] with an average seniority of 20.9 years in the company [min = 1, max = 40]) were suspected of exposure to organic solvents. The work station of the dissolved adhesive consisted of 9 employees (2 men and 7 women), with a 44.6 years median age [min = 25, max = 58] and an average seniority of 23.2 years [min = 6, max = 35]. Ethical consideration was respected in every stage and step of our investigation. The cooperation with this company was within the frame of a voluntary service on solvents risk assessment via a written agreement with the aim of improving conditions in the workshops of the company. Also briefing and information sessions on all study aspects and goals were made in front of the employees before the investigation kick-off. We assured them that all results would be used anonymously with their consent and only for scientific purposes.

### Assessment of exposure to solvents

This study included several facets: (i) an observation of the various work stations; (ii) an investigation with the assistance of the management and the oldest employee on the main modifications which had been taking place overtime, both at the technical level (change of machines, aspiration and ventilation of the buildings,...) and regarding the nature of solvents used; (iii) a retrospective assessment, with the chemical engineer of the company, of the evolution of the solvents' compositions and quantities that are being used; (iv) and finally the current study on personal exposure measurements and bio-monitoring. The latter were carried out in May 2007. To do so, both personal and/or stationary samplings were taken. The personal sampler holder was set near the respiratory person track whereas the stationary sampler was fixed on the earth and kept at the middle height of an ordinary person. Sampling equipments consist of a programmable low flow Pocket Pump (Pump SKC® 210-1002 TX) with a regular flow set to 100 (±5%) cm^3^/mn for 4 hours and an activated charcoal tube 800 and 200 mg (Tube SKC® 226-16) that traps solvents present in the workplace air [11,12]. Once sampled, the tubes were kept under 4°C and transported to the laboratory for the measurement. After solvent desorption in 5 ml of carbon bisulphide, the extract was analyzed using gas chromatography (CPG) with and external calibration mode. The column used is a semi-capillary column HP-5MS (Length = 60 m, Diameter = 0,75 mm) and a flame ionization detection mode (FID). Temperature of the column was firstly set to 43°C to rise till 180°C with a rate of 2°C/min.

Interpretation of the air concentration results was carried out in reference to threshold limit values (TLV) for solvents issued in 3 countries (USA, France and Germany) on the basis of 8 working hours period/day and 39 hours/week (Table 2) [3,13-18]. To account for the combination of solvents we created a cumulative relative index of personal exposure (I.exp) equal to: I.exp = C_1_/TLV_1 _+ C_2_/TLV_2 _+ ...................... + C_n_/TLV_n _Where C_n _and TLV_n _being respectively the measured concentration and the corresponding limit value of a pollutant n. If I.exp is higher than 1, exposure is regarded as excessive. The corresponding index based on stationary work place measurements is called index of pollution (I.pol).

**Table 2:** Workplace air concentrations limit values of studied solvents [1,3,8-10,20,21,23]

	France (EU^1^)		USA (ACGIH^2^)		Germany (MAK^3^)		Our Study (Tunisia)	
**Solvent**	**TLV^4^**		**TLV-TWA^5^**				**Adopted TLV**	
	
	**ppm^6^**	**mg.m^-3^**	**ppm**	**mg.m^-3^**	**ppm**	**mg.m^-3^**	**ppm**	**mg.m^-3^**

Acetone	500	1210	500	-	500	1200	**500**	**1200**
Cyclohexane	200	700	300	-	200	700	**200**	**700**
n-hexane	20	72	50	-	50	180	**20**	**72**
Methylethylketone (MEK, 2-Methyl ethyl ketone)	200	600	200	-	200	600	**200**	**600**
Toluene	50	192	50	-	50	190	**50**	**190**
Trichloroethylene	75	405	50	-	-	-	**50**	**-**

(1) EU: European Union

(2) ACGIH: American Conference off Governmental Industrial Hygienists

(3) MAK: Maximum Arbeitsplatz-konzentration

(4) TLV: Threshold Limit Value to solvents calculated on the basis of 8 work hour period/day and 39 hours/week

(5) TLV-TWA: Time-Werghted Average (Median values balanced over 8 hours per days and 40 hours per weeks)

(6) ppm: shares per million and volume of air

Regarding biological monitoring, 25 volunteering workers participated via urine donations. Nearly 50 ml was collected from each person in clean plastic tubes and immediately kept in a cooling container of about 4°C then transported to the laboratory and either directly analysed or frozen to -20°C. Urine sampling was done twice a week; at the end of the week and at the end of the work station (Thursday afternoon). Metabolites of the studied solvents were quantified using a high performance liquid chromatography (HPLC) [15,18,19]. Separation column was Eurospher-100 C18 type with a length of 250 mm and an internal diameter of 4.6 mm. The mobile phase was a mixture of 900 ml acetic acid solution (2%) and 100 ml methanol. Mobile phase flow rate was set to 1.5 ml under isocratic mode and a detection wave length of 250 nm. All urines samples were firstly acidified with chlohidric acid to a pH around 1, then centrifuged and finally filtered. Measurement was conducted via the injection of an aliquot of 20 μl into the pre-calibrated high performance liquid chromatographic apparatus. Results were compared to different biological exposure limit values (BEI) drawn from the literature (Table 3) [16,20]. Also, since non professional exposure can have an influence both on the renal and hepatic pathology and on the interpretation of the results, an individual information chart was created for each employee on which any other exposure different from the professional one such as leisure activities, smoking or any other aspects was specifed.

**Table 3:** Biological exposure limit values of studied solvents [8-10,19,20,22-24]

		France (EU)	USA (ACGIH)	Germany (DFG^1^)	Our Study (Tunisia)
**Biological Exposure index** **(BEI) in Urine**		**FGV^2^** (1997)	**BEI^3^**	**BAT^4^**	**Adopted** **BEI**

Acetone		100 mg/l	50 mg/l	80 mg/l	**50 mg/l**
1,2-cyclohexanediol		-	-	170 mg/g creat	**170 mg/g creat**
2,5 hexanedione		-	0.4 mg/l	-	**0.4 mg/l**
Methylethylketone		-	2 mg/l	5 mg/l	**2 mg/l**
Hippuric acid		2500 mg/g creat	1600 mg/g creat	-	**1600 mg/g creat**
Trichloroacetic acid		100 mg/g creat	80 mg/l [15 mg/l (Proposal 2007)]	**A**	**15 mg/l**

(1) DFG: Deutsche Forchungs-Gemeinschaft (2) FGV: French Guide Value (3) BEI: Biological Exposure Indices (4) BEATS: Bioloischer Arbeitsstoff-Toleranz-Wert (biological values tolerated in professional environment)					

(A)					

**Concentration of **Trichloroethylene **in the air (ml/m^3^)**	**Acid **trichloroacetic **in the urine (mg/l)**				

10	20				
20	40				
30	60				
50	100				

In the absence of Tunisian exposure limit values for workplace air or biological monitoring [8-10] and as mentioned above, we referred to the French, American and/or German values and adopted the most severe among them (Table 2 and table 3) [3,12,13,21].

## Results

In total, ten air samplings, five personal and five stationary as well as 25 urine samplings were carried out. Table 4 summarizes the main results of the air measurements (number of samples, average values and ranges). It can be seen that air concentrations in the workshop of the dissolved adhesive, in the control laboratory and in the storage halls of the finished products were greater than the TLVs (indexes are above 1; Table 4). Higher concentrations of hexane were also found in the solvented adhesive workshop (respective averages and ranges, in the control laboratory and in the storage rooms were 539.8 mg/m^3 ^[222.0-1415.0], 140.5 mg/m^3 ^[80.0-201.0] and 80.2 mg/m^3^). However, toluene concentrations did not exceed the limit value (respective averages and ranges in the three above mentioned locations were 122.0 mg/m^3 ^[81.0-154.0], 56.0 mg/m^3 ^[51.0-61.0] and 102.0 mg/m^3^). The other solvents, cyclohexane, methylethylketone and trichloroethylene had lower concentrations, in comparison with the TVS. As for benzene it was undetectable.

**Table 4:** Exposure indices according to the workshop

Type of workshop	*Personal exposures*			*Environmental samplings*	
	
	Index of exposure			Index of pollution	
	**N**	**range**	**average**	**N**	**average**
Solvented adhesive workshop	4	4.09 - 20.1 3	8.40	-	-
Non-solvented workshops (*natural adhesive, vinyl adhesive, latex adhesive*)	-	-	-	3	0.01^£ ^
Analysis laboratory	1	-	3.12	1	1.49
Finished products store	-	-	-	1	1.70

	**Total = 5**			**Total = 5**	

***Personal exposure*: Index of exposure **(I.exp)/***Environmental sampling*: Index of pollution **(I.pol)

I.exp = C_1_/TLV_1 _+ C_2_/TLV_2 _+ ...................... + C_n_/TLV_n _

C_n _and TLV_n _being respectively the measured concentration and the limit value of pollutant n. ^£ ^all three index values were 0.01.

Table 5 shows the results for the urine samples. They revealed that concentrations of the 2,5-hexanedione and hippuric acid, respectively biomarkers of hexane and toluene, were high in the workshop of dissolved adhesive. Also high concentration of the 2,5-hexanedione was observed in certain employees' urines working in the vinyl adhesive workshop, in the latex adhesive workshop and in the storage rooms. Similarly, high values of the 2,5-hexanedione were recorded in the urines of those working in the control laboratory with an average of 1.14 mg/l). It is worth noting that in this location, the 2 technicians were handling extremely limited quantities of adhesive samples, and collective and individual protective equipments were available.

**Table 5:** Urine concentrations of solvents metabolites according to the activity of the workshop

Type of workshop		Acetone (mg/l)		2,5 hexandione (mg/l)		Hippuric acid (mg/g créat)		Metylethylketone (mg/l)		Trichloracetic acid (mg/l)	
						
	N	range	average	range	average	range	average	range	average	range	average
Solvented adhesive workshop	9	2.30 - 20.40	6.87	0.12 - 0.98	0.46	92 - 1850	1248	0.20 - 3.80	1.22	4.00 - 21.00	7.67
vinyl adhesive workshop	5	3.30 - 5.20	3.80	0.25 - 1.56	0.99	111 - 742	383	-	< 0.2	0.01 - 0.04	0.02
Natural adhesive workshop	3	2.00 - 6.70	4.23	0.10 - 0.52	0.26	85 - 860	350	-	< 0.2	0.01 - 0.20	0.08
latex adhesive latex workshop	4	4.10 - 5.20	4.58	0.10 - 0.89	0.50	128 - 467	299	-	< 0.2	0.01 - 0.05	0.03
Quality control laboratory	2	6.20 - 7.90	7.05	0.64 - 1.64	1.14	186 - 360	273	-	< 0.2	0.01 - 1.04	0.53
storage of the finished products	2	3.70 - 5.00	4.35	0.24 - 1.14	0.69	45 - 513	279	-	< 0.2	-	0.04

	**Total = 25**										

**N: **number of employees -: not manipluled in the workshop

We noted that the workshop of the dissolved adhesive consists of 2 communicating floors as shown in Table 6. It has natural ventilation with 2 doors, 6 windows and mechanical ventilation. In its 4 working stations (turbines, manufacturing lines on the 1^st ^floor and in the three conditioning stations on the ground floor), the levels of hexane surpassing both the air and biological limit values were noted, mainly at the station of the turbines. In these locations also, we noticed that in spite of the presence of general and individual protective equipments available for all the employees (specific masks and gloves), only 10% were using them.

**Table 6:** Concentrations of hexane and its biological indicator in the workshop of the solvented adhesive


			Hexane concentration		2,5 hexandione Concentration (mg/l)	
			
Location	Stations	Operators	mg/m^3^	ppm	range	average
***1st floor***	Turbines	2 men	1415.0	418.2	0.60 - 0,98	0.79

***Ground floor***	Conditioning 1	3 women	233.0	68.9	0.17 - 0,80	0.38
	Conditioning 2	2 women	222.0	65.6	0.12 - 0,40	0.26
	Conditioning 3	2 women	289.0	85.4	0.38 - 0,51	0.45

***Average in the workshop***			***539.8***	***159.5***	***0.12 - 0.98***	***0.46***

C_i_: Conditioning ●: permanent agent: : reinforcement agent

## Discussion

This study reports that workplace air and personal exposure concentrations of several VOCs are exceeding threshold limit values set at developed industrialized countries. The solvented adhesive workshop was where exposures were the greatest, namely for hexane and its biomarker the 2,5-hexandione.

Several studies have discussed how utilisation of solvents has gone through a significant great evolution as well as prevention measures and regulations in solvented workplaces. This evolution has also touched the modifications of solvents nature and the way they are handled [3,11]. However, it was reported that the number of workers exposed to solvents has been increased. For instance in France, the SUMER 2003 study shows that between 1994 and 2003, the proportion of exposed workers passed from 12.2% to 14.7%, especially in the chemical industry [1].

In addition, risk assessment studies have been conducted in various industrial sectors. However, the adhesive manufacturing area has been somehow neglected [3]. To the best of our knowledge, this is the first exposure study ever conducted in the adhesive manufacturing sector in Tunisia. This work also included a qualitative appreciation of the risk by a careful investigation of the working stations and an inventory of the products that were handled. Environmental and biological measurements were performed in our study in the framework of an effort to assess their impact on humans [22,23].

As was mentioned above, faced with the absence of data in the literature dealing with developing countries comparable to Tunisia, we referred to data used in the more developed world. Found results revealed that the cumulative indexes of exposure were much higher than 1 in particular in the three studied locations, with greatest values in the solvented adhesive workshop. Indeed an average of 8.40 was noted in this location; meanwhile the lowest value (4.09) was recorded in one of the conditioning stations and the maximum value of 20.13 at the turbines stations. In Washington DC (USA), in an aerospace manufacturing company the average (range of observed values) cumulative exposure index for VOCs among painters was 3.77 (0.20-10.6) [20]. In Japan, during the summer season of 1999, the average of solvent exposure index in furniture factories was 0.35 (maximum = 5.35); distinctively for hexane. Also, the mean concentration was 1.0 mg/m^3 ^with a maximum of 45.5 mg/m^3 ^for the same solvent [21].

Whereas in Spain, according to Cardona [24,25], the average values of hexane and toluene among shoe factory workers (individual exposure), were respectively 47 mg/m^3 ^and 86 mg/m^3^, and ranging respectively between 4 to 652 mg/m^3 ^and 2 to 1143 mg/m^3^. In Italy, under almost the same working environment conditions, Baldasseroni [26] found an average individual value of 0.86 mg/l for the 2.5-hexandione, close to what we found in the solvented adhesive workshop and which was equal to 0.46 mg/l. In California, Wilson MP et al. [27] conducted a study on vehicle repair industry where aerosol products containing hexane, toluene, acetone and methyl ethyl ketone were used extensively. They reported an average (range) of hexane and toluene air-concentrations in vehicle repair shops of 28 mg/m^3 ^(2 to 100) and 11.2 mg/m^3 ^(2 to 21), respectively. Another study showed that in 2004 [28], in a French serigraphy company, the median atmospheric concentration of solvents was 80.1 mg/m^3 ^for benzoic hydrocarbons. In the Netherlands, Hertsenberg et al [29] found average hexane and toluene concentrations respectively of 25.7 and 26.0 mg/m^3 ^in shoe repair shops during 2005. Average urinary concentration of 2,5-hexandione was 3.2 ± 2.9 mg/l among shoe makers in Turkey in 1997 [30], and the mean of hexane was 411.6 mg/m^3^.

In the current study, high values of 2,5-hexanedione (metabolite of hexane) were observed in the urines of the employees working in the vinyl adhesive workshop. Recently, air concentrations have been lower than the TLVs in this workshop. This can be explained by the fact that these workers are sometimes called to help their colleagues in the solvented adhesive workshop at the conditioning stations in case of important orders. Hexane levels were higher than the reference values in the urines of the 2 technicians. This was a surprising finding since one of the technicians had been working there just for 15 days. Indeed, despite a frequent handling of solvented adhesive for quality control, hexane and other solvents were in tiny quantities in this place. A new visit to the laboratory revealed that cleaning of the laboratory equipments at the end of the station was done with hexane in spite of the rules prohibiting such usage.

As this was the first assessment of exposure to organic solvents, one limitation of this investigation was that it involved only one company but which is considered as having an advanced policy towards work condition improvement and where workers were participating voluntarily. Moreover, although the number of the exposed persons might be small, we believe that this does not affect the authenticity and the findings of the investigation. Indeed, the sampling methodology was conducted to mainly identify where employees are supposed to be exposed to the highest values of organic solvents and not according to the size of the company manpower in concordance with what is reported elsewhere [4,11,12].

Probably, another weakness of this study is that it was conducted during a relatively narrow time period. In fact, the production activity varies throughout the year according to orders and this may affect the working atmosphere quality. Also the natural ventilation of the different workshops is influenced by seasons. For instance, doors or windows are open during the hot period and also at the time of the current investigation.

Interestingly, the types of solvents, and the proportions reported in Table 1 were comparable with those described by Samato *et al*. in Kyoto, Japan (April 2004 to March 2005) [31] where the solvents used in an adhesive industry were toluene (47%), ethyl acetate (42,5%), MEK (33%), acetone (27%) with lower usage of trichloroethylene.

Finally we note that neuropsychiatry symptoms associated with chronic exposure to organic solvents have been described for a long time. The effects of chronic and sub-chronic exposure to these studied organic solvents on balance control of our volunteer workers have been published in a previous paper [32].

## Conclusion

Although regulations exist, pollution biomonitoring is not yet compulsory either in Tunisia or other countries alike. We recall that we conducted a regional survey and had spent almost 10 months gathering information on all adhesive manufacturers and users in this region to finally carry out this study with this cooperating company. Therefore, it can be concluded that this study establishes the first report on the profile of occupational exposure to organic solvents used in the Tunisian adhesives producing industries. High exposure levels were recorded in the studied company which is likely to offer better work conditions compared to the many others of the country. Nonetheless, it is worthwhile to replicate the current investigation in other parts of the country and set a preliminary data base for the Tunisian Work Health Association.

## Competing interests

The authors declare that they have no competing interests.

## Authors' contributions

**IG **was involved in the conception and design of the project, data collection, data analysis, data interpretation, manuscript writing, and final approval of manuscript. **MK **was involved in the conception and design of the project, data collection, data analysis, data interpretation, manuscript writing, and final approval of manuscript. **CN **was involved in the conception and design of the study and data interpretation. **AL **participated in the design of the study and performed the statistical analysis. **MLM **contributed to the data interpretation. **PF **was involved in the data interpretation. **DM **contributed in the data interpretation. **BE **contributed to the conception and design of the project, data collection, data interpretation, manuscript writing, and final approval of manuscript. **DZN **was involved in the conception and design of the project, data interpretation, manuscript writing, and final approval of manuscript. All authors read and approved the final manuscript.
